# Supplementary material for: Resolving the full spectrum of human genome variation using Linked-Reads
Source: Genome Res. 2019 Apr;29(4):635–45. doi: 10.1101/gr.234443.118 (PMC6442396; doi:10.1101/gr.234443.118)
Supplement: Supplemental Material [file supp_gr.234443.118_Supplemental_longranger-2.2.zip › longranger-2.2.tar.bz2!/lr2.2-public/lib/stan/cmdstan/stan/doc/api/html/structstan_1_1gm_1_1print__statement.html]

Stan: stan::gm::print\_statement Struct Reference


|  |  |
| --- | --- |
|  | Stan  2.5.0  probability, sampling & optimization |


- Main Page
- Related Pages
- Namespaces
- Classes
- Files

- Class List
- Class Index
- Class Hierarchy
- Class Members

All Classes Namespaces Files Functions Variables Typedefs Enumerations Enumerator Friends Macros Pages

- stan
- gm
- print\_statement

Public Member Functions |
Public Attributes |
List of all members

stan::gm::print\_statement Struct Reference

`#include <ast.hpp>`

|  |  |
| --- | --- |
| Public Member Functions | |
|  | print\_statement () |
|  | |
|  | print\_statement (const std::vector< printable > &printables) |
|  | |

|  |  |
| --- | --- |
| Public Attributes | |
| std::vector< printable > | printables\_ |
|  | |

## Detailed Description

Definition at line 745 of file ast.hpp.

## Constructor & Destructor Documentation

|  |  |  |  |  |
| --- | --- | --- | --- | --- |
| stan::gm::print\_statement::print\_statement | ( |  | ) |  |

Definition at line 1329 of file ast\_def.cpp.

|  |  |  |  |  |  |
| --- | --- | --- | --- | --- | --- |
| stan::gm::print\_statement::print\_statement | ( | const std::vector< printable > & | *printables* | ) |  |

Definition at line 1331 of file ast\_def.cpp.

## Member Data Documentation

std::vector<printable> stan::gm::print\_statement::printables\_

Definition at line 746 of file ast.hpp.

---

The documentation for this struct was generated from the following files:

- src/stan/gm/ast.hpp
- src/stan/gm/ast\_def.cpp


---

[ Stan Home Page ]

*© 2011–2014,
Stan Development Team.*
